# The relationship between depression and cognitive function in adults with cardiovascular risk: Evidence from a randomised attention-controlled trial

**DOI:** 10.1371/journal.pone.0203343

**Published:** 2018-09-05

**Authors:** Haley M. LaMonica, Daniel J. Biddle, Sharon L. Naismith, Ian B. Hickie, Paul Maruff, Nicholas Glozier

**Affiliations:** 1 Brain and Mind Centre, University of Sydney, Camperdown, Australia; 2 Charles Perkins Centre, School of Psychology, University of Sydney, Camperdown, Australia; 3 Central Clinical School, Sydney Medical School, University of Sydney, Camperdown, Australia; 4 Cogstate, Melbourne, Australia; North Carolina Neuropsychiatry Clinics, UNITED STATES

## Abstract

**Background and aim:**

This study assessed the association between depressive symptom severity and cognition in middle-to-older aged adults with mild-to-moderate depression and cardiovascular risk factors using an online test battery (*CogState*) and whether changes in depressive symptoms over 3 months were associated with changes in cognition.

**Methods:**

Participants (mean age = 57.8) with cardiovascular risk and mild–to-moderate depressive symptoms completed measures of psychomotor speed, learning, and executive function prior to (n = 445)_and after (n = 334) online depression or attention control interventions. The symptom severity-cognition relationship was examined both cross-sectionally and prospectively.

**Results:**

Participants exhibited significantly reduced psychomotor speed and variable impairments on measures of learning and executive functioning relative to normative data. However, there was no association of depression severity with cognition at baseline or of change in depressive symptoms with change in cognitive performance.

**Limitations:**

Participants were well-educated, which may have protected against cognitive decline. Attrition may limit generalisability, though is unlikely to explain the lack of association between depression symptoms and cognition.

**Conclusions:**

Adults with comorbid mild-to-moderate depressive symptoms and cardiovascular risks performed less well than age-matched normative data on three online cognitive tests; however, we were unable to show any symptom-cognition association cross-sectionally or longitudinally, despite significant improvements in depressive symptoms. This challenges the generalisability of such associations found in more severely unwell clinical samples to those with a broader depressive symptom profile, or suggests that underlying cardiovascular disease may account for the association seen in some clinical studies. This has implications for scaling up selective prevention of cognitive decline.

## Introduction

It is recognized widely that Major Depressive Disorder (MDD) is associated with cognitive dysfunction [1–3] including impaired learning, working memory, processing speed, and executive functions [4–6]. The neuropsychological profile is, however, heterogeneous and varies with depressive symptom severity [7, 8], disease subtype [9], age of onset [10], etiology, co-morbidities and cerebrovascular disease (CVD) [8]. Regardless of the contributing factors, cognitive dysfunction in depression is associated with substantial disability and poorer quality of life [11], and frequently has been suggested as a selective target for interventions aimed at preventing cognitive decline and subsequent dementia [12].

There are three key issues that might limit such a strategy:

1. Is the depression severity-cognition association present only in the small group with diagnosed MDD or is it applicable to the much larger group with mild to moderate depression symptoms?
2. Does improvement in depression result in a significant improvement in cognitive function? The observed cognitive dysfunction of MDD often persists despite reductions in depressive symptoms [13, 14], and any improvements may not reach the levels of cognition that existed prior to onset of the depressive episode; the “scar hypothesis” [13, 15, 16]. Up to 75% of older adults whose symptoms of MDD have reduced following therapy with antidepressant medication can still be classified as having subtle cognitive dysfunction [17].
3. Is the observed association between depression and cognition a reflection of an underlying common cause such as Cerebrovascular Disease (CVD)? ‘Vascular depression’ describes a syndrome whereby depression occurs for the first time in later life, associated with underlying CVD, marked cognitive impairment and poor prognosis. In this instance, CVD is thought to cause structural changes to CNS white matter [18] which themselves give rise to slowed processing speed, poor memory and executive dysfunction as well as an increased likelihood of progression to dementia [8]. Thus, any examination of depression and cognition in middle-to-older aged adults needs to take into account the presence of CVD and associated vascular risk factors.

Given these questions, we undertook a secondary analysis of a large trial of an online treatment that was shown to be effective for depressive symptoms in community-based adults with mild-to-moderate depression symptoms and self-reported history of CVD or cardiovascular risk factors who underwent cognitive testing at baseline and post intervention. We hypothesised that the depressive symptom severity-cognition association would be found in this group, and that improvements in depressive symptoms would be associated with improvement on cognitive testing despite the presence of cardiovascular risk for cognitive dysfunction.

## Materials and methods

### Study design

The protocol of the Cardiovascular Risk and E-Couch Depression Outcome (CREDO) trial has been published elsewhere [19] and the primary results of the CREDO study have been published [20]. CREDO was a double-blind, parallel group randomised controlled trial designed to compare the effectiveness of internet cognitive behavioural therapy (eCBT) relative to an online attention control in adults, aged 45- to 75-years, with self-reported CVD or significant CVD risk factors, and repeated evidence of mild–to-moderate depressive symptoms.

The trial complied with the Code of Ethics of the World Medical Association. Ethical approval was obtained from the University of Sydney Human Research Ethics Committee in June of 2009 and from the Australian National University Human Research Ethics Committee in 2010. Additionally, the trial was registered with the Australian and New Zealand Clinical Trials Registry (ACTRN12610000085077). The *45 and Up Study* had primary ethical approval from the University of New South Wales Human Research Ethics Committee.

### Participants

Participants (n = 562) were recruited through the *45 and Up Study*, a large-scale longitudinal population-based cohort study comprising over 260,000 men and women aged 45-years and over in New South Wales (NSW), Australia [21]. Comparative analyses between the *45 and Up Study* and the NSW Population Health Survey revealed that the estimated relative risk of a range of health-related risk factors generalised to the wider population [22]. From July 2010 and January 2011, participants were randomly selected from the *45 and Up Study* database using an algorithm to identify those that met the eligibility criteria described in detail in Glozier et al. (2013). In summary, participants were required to have provided a valid email address and to have a self-reported history of CVD or CVD risk factors and persistent or recurrent symptoms of depression. Following an online informed consent process, participants completed online baseline assessments and were randomised to either intervention arm using a customised, fully automated randomisation facility built into the trial website. Randomisation was stratified by depressive symptom severity. Participants were blinded to which programme was the ‘active’ intervention.

### Interventions

The “active’ intervention was E-couch, an automated software program that offers 12 modules addressing mental health literacy, cognitive behavioural therapy (CBT), interpersonal therapy (IPT), relaxation techniques and exercise programs targeting depression. This has been demonstrated to be effective compared to an attention control in reducing depressive symptoms in younger groups without comorbidities [23].

The attention control was HealthWatch, a 12-week online program in which participants were provided with information on a variety of topics including nutrition, physical activity, heart health, and pain. The attention control intervention was matched for contact (i.e., 12 modules) and was used to balance the effects of the expectation of therapeutic benefit offered in the active treatment arm. In previous trials, participants showed a small, although significant reduction in depressive symptoms using this program, potentially reflecting natural remission or some small effect [24].

### Measures

Depressive symptoms, the primary pre-specified outcome measure for the trial, were measured using the PHQ-9 at baseline, 4-, 8-, and 12-weeks. The PHQ-9 is a nine item measure of DSM depression symptoms which has been found to be a reliable and valid screening measure, is widely used in previous community studies of people with depression, and is sensitive to change in clinical status [25, 26].

Three aspects of cognition were assessed at baseline and 12 weeks using an online version of *Cogstate*, a computerised neuropsychological test battery with established validity and sensitivity for the detection of subtle cognitive change in community cohorts in general and also in MDD [27]. For this trial, an internet version was utilised, which was downloaded onto participants’ computers to prevent bandwidth and connectivity interference. The test battery was chosen for brevity as well as capacity to assess neuropsychological skills commonly affected in older people with depression [3, 4]. The *Detection Task* required participants to respond when a card presented on the screen turned over the course of two minutes. Speed was the primary outcome, with lower scores reflecting better performance. Visual learning and memory was assessed with the *One Card Learning Task*. During this five minute, one-back style task, participants responded ‘yes’ or ‘no’ if a card presented on-screen was the same as a previous card. The score reflects accuracy of performance. Executive functioning was assessed with the *Groton Maze Task*. In this five minute, spatial problem-solving task, participants were shown a grid of tiles on-screen and had to use the mouse to find a hidden pathway based on trial and error feedback. The score reflects the number of errors, again with lower scores reflecting better performance.

As part of their baseline assessment through the *45 and Up Study*, participants completed a questionnaire about demographic and social characteristics, personal health behaviours, and general health-related data [21]. These data, the coding of which is standard and detailed in [20] were included in the present study for the purposes of characterising the sample and to assess attrition bias and potential confounding.

### Analysis

Descriptive statistics were used to summarise the baseline demographic and health characteristics of the study sample, and their association with the measures of cognition. One-sample t-tests were then used to compare performance on the cognitive tests between the CREDO sample and age-matched normative data based on a healthy population of subjects enrolled in clinical trials as well as research and academic studies [28].

#### Hypothesis one: Depressive symptom severity-cognition association

Cognitive function values were then converted to normalised scores based on the above-mentioned age-matched normative data [28], and Spearman correlations were used to estimate the relationship between depression severity and cognition at baseline. Pearson product-moment correlations were used to assess the relationship between normally-distributed cognitive and binary sociodemographic and clinical characteristics. Linear regression using the enter method was also used to explore this relationship adjusting for potential confounders. At step 1 depression was entered alone, at step 2 age, gender, education (post-school versus no post-school), psychotropic medication, and intervention arm were entered, and finally diagnosed and treated cardiovascular disease was entered at step 3.

Before conducting linear regression models, major assumptions of the approach were assessed. Homoscedasticity and normality of residuals was confirmed through inspection of scatterplots and graphs, respectively. Lack of association between independent variables and residuals was confirmed through examining correlations for continuous independent variables and t-tests for binary independent variables. Potential multicollinearity was assessed via examining variance inflation factors (VIFs). As residuals for the cross-sectional analysis of executive function were non-normal, executive function was further transformed into its natural logarithm for this analysis.

Outliers, defined as cases scoring +/-3 standard deviations from the mean on the outcome or independent variable of interest for univariate analyses (t-tests and correlations), and standardized residuals <-3 or >3 for multivariate analyses (regressions), were excluded.

#### Hypothesis two: Change in cognitive function

For those with baseline and follow-up cognitive function data, paired t-tests were used to assess change in cognitive variables and depression over the course of the study. Change scores were then calculated for each normalised cognitive function variable, along with depressive symptoms, by subtracting follow-up scores from baseline scores. Linear regression was used to estimate associations between changes in depressive symptoms and changes in cognitive function and then adjusted, in a stepwise fashion as above, for age, gender, education, depression symptoms, medication and intervention arm, and then the presence of diagnosed CVD and risk factors. Outliers (cases with standardized residuals <-3 or >3, were excluded from these analyses. In sensitivity analyses the regression models were re-run with MDD, current depression symptom severity (centered), and their interaction, entered at step 1, to gauge whether the depression-cognition relationship was apparent for those with probable MDD at baseline. Probable MDD was defined as scores of 2 or more on either questions 1 or 2 of the PHQ-9, and a total of 5 or more items with scores of 2 or 3 [29]. In a further sensitivity analysis designed to take into account status at baseline, latent change scores were created for depression and cognitive function tasks by regressing status at baseline on status post-intervention. The residuals, which equate to the estimated change in each variable not explained by status at baseline, were entered into regression models in the same manner described above. Results are reported such that positive coefficients equate to better cognitive performance or improvement (i.e. faster reaction times, greater learning accuracy, fewer executive function task errors).

## Results

As referenced above, the primary results of the CREDO trial have been published previously [20]. For the purposes of this study, the data from both intervention arms was pooled.

### Baseline demographics and health characteristics

Of the 562 recruited participants, 445 (79.2%) completed at least one baseline cognitive task. Baseline characteristics for these participants are presented in Table 1. Participants had a mean age of 58-years. The majority of participants were female (63.6%), spoke English at home (94.8%), and had more than a high school level of education (73.2%). Over half the sample (55.5%) had a prior diagnosis of depression. At baseline, the mean PHQ-9 score was 11.93 (SD: 3.4) and 24% (n = 107) of the participants met criteria for probable MDD. There were no statistically significant differences in any variable between arms indicating adequate randomisation [20]. There were few significant differences between those who completed at least one baseline cognitive task and those who did not. Those with baseline cognitive data were more likely to be female (63.6% vs 53.0%), partnered (74.5% vs 64.0%), taking psychotropic medication (35.96% vs 25.86%) and younger (M = 57.54 vs 59.48), than those who did not complete any baseline cognitive measures.

**Table 1:** Baseline characteristics of 445 CREDO participants, and their association with baseline cognition.

Characteristics		Psychomotor speed	Learning	Executive function
**Continuous Measures**	**Mean (SD)**	**ρ**	**ρ**	**ρ**
Age in Years	57.8 (6.6)	.194***	-.104*	.123**
Depression (PHQ-9)	11.93 (3.4)	.038	.014	-.027
**Categorical Measures**	**N (%)**	**r**	**r**	**r**
Sex: Female	283 (63.6)	.007	.070	-.029
Sex: Male	162 (36.4)			
English spoken at Home: Yes	422 (94.8)	-.013	.030	-.017
English spoken at Home: No	23 (5.2)			
Marital Status: Partnered	332 (74.6)	-.094*	.067	-.020
Marital Status: all others	113 (25.4)			
Highest Qualification: Post-school +	325 (73.2)	.069	.077	.067
Highest Qualification: high school or less	119 (26.8)			
Private Health Insurance: Yes	289 (64.9)	.053	.050	.040
Private Health Insurance: No	156 (35.1)			
Probable MDD [29]: Yes ++	107 (24.0)	.062	.032	-.045
Probable MDD [29]: No ++	338 (76.0)			
Prior diagnosis of Depression: Yes	247 (55.5)	.009	-.040	.049
Prior diagnosis of Depression: No	198 (44.5)			
Prior diagnosis of Anxiety: Yes	148 (33.3)	.047	.044	-.019
Prior diagnosis of Anxiety: No	297 (66.7)			
Psychotropic medication: Yes	160 (36)	-.081	-.075	-.061
Psychotropic medication: No	285 (64)			
Prior diagnosis Cardiovascular Disease: Yes ~	310 (69.7)	-.082	-.024	.017
Prior diagnosis Cardiovascular Disease: No ~	135 (30.3)			
Treatment for any CVD in Last Month: Yes #	309 (69.4)	-.072	-.086	-.107*
Treatment for any CVD in Last Month: No #	136 (30.6)			
Family History of CVD: Yes	297 (66.7)	.034	-.028	-.016
Family History of CVD: No	148 (33.3)			
Hazardous drinker [30]: Yes ^	169 (47.3)	.082	.047	.084
Hazardous drinker [30]: No ^	188 (52.7)			
Current Smoker: Yes	68 (15.3)	.093	-.025	-.074
Current Smoker: No	377 (84.7)			
Obese (BMI>30): Yes	205 (47.6)	.008	-.002	-.041
Obese (BMI>30): No	226 (52.4)			
Exercise—Sufficient Time and Sessions: Yes $	227 (51.0)	-.022	-.105*	-.027
Exercise—Sufficient Time and Sessions: No $	218 (49.0)			
One or more Comorbid Conditions: Yes ##	248 (55.7)	-.028	-.087	.037
One or more Comorbid Conditions: No ##	197 (44.3)			

^+^ n = 1 missing education.

^++^ Probable MDD was defined as scores of 2 or more on either questions 1 or 2 of the PHQ-9, and a total of 5 or more items with scores of 2 or 3.

^~^ Prior Diagnosis of Cardiovascular Disease includes doctor diagnosis of any one of Heart Disease, Stroke or Hypertension.

^ Optimal screening cut points for the AUDIT-C, a 3-item alcohol screening questionnaire, for alcohol dependence based on a sample of individuals with a history of psychopathology in the past year. NB n = 88 missing AUDIT scores.

^$^ At least 150 mins of self-report moderate activity over at least 5 sessions each week

^#^ Treatment for any Cardiovascular Disease includes: any one of heart attack/angina, other heart disease hypertension or high blood cholesterol.

^##^ Other comorbid conditions include: cancer (skin, prostate, breast or other cancer), blood clot (thrombosis), asthma, Parkinson’s disease, osteoarthritis, and/or thyroid problems.

* p < .05.

** p < .01.

*** p < .001

NB 4 participants did not complete psychomotor speed task (DET) at baseline. Outliers (cases with scores scores +/- 3 SD from the mean) were excluded from correlations for continuous variables psychomotor speed (n = 6), visual learning and memory (n = 5), executive function (n = 3), and depression (n = 2).

Although cognitive function was worse in the older participants (slower psychomotor speed (ρ = -.194, p <.001), worse learning (ρ = -.104, p <.05), and worse executive function (ρ = -.123, p <.01)) there were few other associations of demographic, illness or social variables with the cognitive function measures. Of the 54 associations analysed in addition to age, only three were associated, and then with only one of the measures each, a number quite likely due to chance.

### Cognitive function in mild-to-moderate depression compared to normative data

Compared to aged-matched groups from the *CogState* normative data (Table 2), the study participants showed a significant impairment in psychomotor speed in each age group. Similar results were seen in relation to learning accuracy, with significant deficits noted for participants aged 50 to 59 and 60 to 69, but not for those in their 70s. Those in their 60s made fewer errors on the executive functioning tasks than age-matched peers, however when outliers (n = 2) we included this difference was no longer significant. There were no significant differences in performance level on the executive functioning task for those subjects in their 50s, and 70s relative to age-matched peers. A one-way ANOVA suggested significant differences in depressive symptoms as a function of age (F(3,439) = 5.396, p = .001), and post-hoc contrasts indicated participants 70–79 years of age had less depressive symptoms at baseline than those 50–59 years of age (t = 2.15, p = 0.032).

**Table 2:** Participant cognitive test performance compared to Cogstate normative reference data, and participant baseline depressive symptoms by age group.

Outcome Measure	Source (n)	Age Group	Mean (SD)	t	p-value
Psychomotor function: speed of performance on Detection Task	CREDO (232):	50–59 yrs	2.58 (0.10)	7.99	<0.001
	Normative (461):		2.53 (0.11)		
	CREDO (135):	60–69 yrs	2.62 (0.10)	8.73	<0.001
	Normative (561):		2.54 (0.11)		
	CREDO (17):	70–79 yrs	2.63 (0.10)	2.82	0.012
	Normative (400):		2.56 (0.11)		
Learning: accuracy of performance on One Card Learning Task	CREDO (235):	50–59 yrs	0.97 (0.09)	-8.67	<0.001
	Normative (206):		1.02 (0.11)		
	CREDO (137):	60–69 yrs	0.97 (0.09)	-7.05	<0.001
	Normative (575):		1.02 (0.11)		
	CREDO (16):	70–79 yrs	0.97 (0.10)	-1.04	0.316
	Normative (531):		1.00 (0.10)		
Executive function: total number of errors made in attempting to learn the same hidden pathway on five consecutive trials at a single session on Groton Maze Task	CREDO (237):	50–59 yrs	55.34 (22.66)	-0.22	0.828
	Normative (298):		55.70 (28.78)		
	CREDO (136):	60–69 yrs	55.93 (23.50)	-3.15	0.002
	Normative (329):		62.29 (23.09)		
	CREDO (17):	70–79 yrs	77.12 (40.19)	1.30	0.212
	Normative (275):		64.44 (26.22)		
PHQ-9 continuous: depressive symptom severity	CREDO (52):	45–49 yrs	12.13 (3.67)		
	CREDO (238):	50–59 yrs	12.47 (3.71)		
	CREDO (138):	60–69 yrs	11.09 (2.66)		
	CREDO (17):	70–79 yrs	10.59 (3.04)		

Outliers (cases with scores scores +/- 3 SD from the mean) were excluded from these analyses for psychomotor speed (n = 6), visual learning and memory (n = 5), executive function (n = 3), and depression (n = 2). Comparisons with cognitive function norms for the 45-49-year-olds are not displayed as norms were only available for those 35–49 years of age.

### Course of depressive symptoms and cognition over the study

Over the 12 weeks of the study, participants in both arms showed improvements in depressive symptoms of 3.66 points (95% CI: 3.05–4.27) with eCBT and 2.60 points (95% CI: 2.05–3.16) in the control group, with a significantly greater decline in the PHQ-9 for eCBT compared to control (1.06; 95% CI: 0.23–1.89; time by arm interaction p = .012).

The complete results relating to the effects of the web-based intervention on depression symptom severity were published previously [20]. Of participants with baseline cognitive function data (n = 445), 75% (n = 334) completed at least one of the cognitive measures at week 12 (see Table 3). Those who were missing week 12 cognitive data had lower baseline scores for the learning task (M = -.59, SD = .76) than completers (M = -.37, SD = .79, t(443) = -2.623, p = .009), and worse scores for executive function (M = -.05, SD = .95) than completers (M = .16, SD = .89, p = .035), but did not differ in baseline psychomotor speed or PHQ-9 scores. Those who provided only baseline data did not differ on most characteristics, but were less likely to speak English at home (89.2% vs 96.7%, X^2^ = 9.606, p = .002) and be obese (56.9% vs 44.4%, X^2^ = 5.078, p = .024), neither of which showed any association with baseline cognition. Of the cognitive measures only executive function showed any change over the 12 weeks of the study.

**Table 3:** Cognitive test scores and depressive symptoms over the study and paired t-tests assessing significance of change for those with baseline and follow-up data for at least one measure of cognitive function.

Cognitive test	Baseline	Post intervention	Change		
	Mean (SD)	Mean (SD)	Mean (95%CI)	t	p-value
Psychomotor speed (n = 319)	-0.60 (1.07)	-0.58 (1.04)	+ 0.01 (-0.09, 0.11)	0.22	0.826
Visual Learning and memory (n = 327)	-0.36 (0.80)	-0.29 (0.78)	+ 0.07 (-0.15, 0.01)	1.70	.089
Executive function (n = 331)	-0.16 (0.89)	0.35 (0.83)	+ 0.19 (0.09, 0.28)	3.87	<.001
Depression (PHQ-9) (n = 330)	11.93 (3.42)	8.78 (4.70)	- 3.15 (-2.67, -3.62)	13.05	<.001

Psychomotor speed = CogState Detection task (DET) z-score (normalized)–more negative score = slower reaction time, Learning = CogState One Card Learning task (OCL) z-score (normalized)–more negative score = poorer performance, Executive function = CogState Groton Maze Learning task (GML) z-score (normalized)–more negative score = more errors, Depression = PHQ-9 score. NB 8 participants completed all week 12 data except for psychomotor speed, and outliers (cases with change scores +/- 3 SD from the mean) for psychomotor speed = 7, visual learning and memory = 7, executive function = 3, and depression = 4.

### Cross-sectional association of depression severity, and cognition

Baseline depression severity was not associated with performance on any of the cognitive tasks, with correlations ranging from -.038 to .027, and p-values from .425 to .768 (see Table 1). In the linear regression models there were no associations between depression severity and cognitive function either with or without adjustment for potential confounders (see Table 4).

**Table 4:** The cross-sectional association of cognitive function and depressive symptoms at baseline, and association of change in cognitive function and change in depressive symptoms.

	Step 1			Step 2			Step 3		
**Baseline analysis**	***B***	**95%CI**	**t**	***B***	**95%CI**	**t**	***B***	**95%CI**	**t**
Psychomotor speed (DET)—Depression	-0.02	-0.03, 0.02	0.39	0.00	-0.03, 0.03	0.07	0.01	-0.03, 0.03	0.15
Learning (OCL)–Depression	0.01	-0.08, 0.10	0.22	0.01	-0.08, 0.11	0.24	0.01	-0.08, 0.11	0.24
Executive function (GML)—Depression	-0.01	-0.16, 0.03	1.36	0.00	-0.15, 0.05	0.99	0.00	-0.14, 0.05	0.95
**Longitudinal analysis**	***B***	**95%CI**	**t**	***B***	**95%CI**	**t**	***B***	**95%CI**	**t**
Δ Psychomotor speed (DET)—Δ Depression	0.09	-0.02, 0.20	1.56	0.10	-0.01, 0.21	1.75	0.10	-0.01, 0.21	1.72
Δ Learning (OCL)—Δ Depression	0.02	-0.09, 0.13	0.31	0.02	-0.09, 0.13	0.38	0.02	-0.09, 0.13	0.40
Executive function (GML)—Δ Depression	0.01	-0.09, 0.12	0.27	0.02	-0.09, 0.13	0.40	0.02	-0.09, 0.13	0.40

Note.

*p <.05.

**p <.01.

***p <.001.

Variables entered at Step 1 = depression, Step 2 = age, gender, education, psychotropic medication, treatment arm, Step 3 = cardio-vascular disease. B = standardized beta coefficients. Residual outliers removed for cross-sectional analysis of psychomotor speed (n = 8), learning and memory (n = 5), and executive function (n = 5). Residual outliers also removed for change score analysis of psychomotor speed (n = 7), learning and memory (n = 6), and executive function (n = 3).

### Association of change in cognitive function and change in depressive symptoms

Change in depression symptoms was not associated with change in any of the cognitive function variables (Table 4). There was no effect on this lack of association when adjusting for potential confounders. However, in the sensitivity analysis including an interaction between symptom severity and probable MDD, a significant interaction was observed for change in psychomotor speed at the first step of the model (t = 2.746, p = .006). The model became non-significant when covariates were added, and no covariates were significant predictors of change in psychomotor speed, so the first model (with change in depression, probable MDD, and their interaction) was retained. In a post-hoc split-file regression analysis it was found that only for those with probable MDD was change in depressive symptom severity associated positively with change in psychomotor speed. For these participants, change in depressive symptoms explained 12.6% of the variability in change in psychomotor speed, with each standard deviation increase in depression change associated with a .355 standard deviation increase in psychomotor speed change (t = 3.285, p = .002).

In the latent change sensitivity analysis, the results mirrored those of the initial analysis i.e. no prospective associations between depression and cognitive function were found.

## Discussion

This study suggests that people with mild-to-moderate depressive symptoms and self-reported CVD or cardiovascular risk factors generally perform below an aged matched normative database on measures of psychomotor speed and learning accuracy. Participants aged 60 to 69-years and 70-to-79 years also exhibited executive dysfunction, but there was no evidence of executive dysfunction for those participants in their 50s. Contrary to our hypotheses there was no association at baseline between depression symptom severity and cognitive performance and no suggestion of potential (reverse) confounding by variables including age, gender, education, psychotropic medications, treatment arm, and diagnosed CVD. This null finding suggests that the symptom severity cognition association commonly observed in clinical samples [7] may not be evident in community settings where individuals may have a more heterogeneous and milder pattern of symptoms. Given that cognitive dysfunction is associated with greater disability in depression [2, 11] and that functional impairment can drive help seeking, such a discrepancy between settings might be expected. This does, however, have implications for the generalisation of findings from clinical settings.

As previously reported [20], over the course of the study, participants in both arms showed improvements in depressive symptoms, significantly greater in the active arm, although with a small effect size. However, only executive function improved over the course of the study and there was no association of depressive symptom reduction with cognitive improvement over the 12 weeks overall. Amongst the small group with probable MDD at baseline, the change in depressive symptom severity was associated with change in psychomotor speed, but not in learning or executive functions. These results may indicate a real lack of association between changes in depressive symptom and cognition in this group with predominantly mild-to-moderate depression, especially given there was no association of symptoms and cognition at baseline. The lack of association may also reflect the small size of the change in depression and that of the cognitive measures only executive function showed statistical improvement. However, although the mean changes were small, their marked variance could have reflected intra-individual correlations. It may be that in this study a mean change of only 3 or 4 on the PHQ-9 may be inadequate to lead to observable changes in neuropsychological test performance, or that there may be a time lag between changes in affective symptoms and cognition as has been observed with function [31].

In contrast to previous studies, we did not find that elevated symptoms of depression were associated with greater cognitive dysfunction nor did we find that a reduction in depression symptom severity was associated with cognitive improvement [7]. Furthermore, based on available data, we cannot conclude that the relationship between depression symptom severity and cognition was masked by a common cause, such as diagnosed CVD; however, it is possible that unmeasured confounders such as age of onset (early *vs*. late onset depression), duration of illness, episode frequency [3, 32], comorbid anxiety, underlying white matter change or other medical comorbidities may have mediated any relationship between depressive symptom severity and cognition, thus potentially concealing an association in this instance. The suggestion that there was however a symptom-cognition association in those with probable MDD is intriguing. Could it be that the frequently observed association is only present in those with a “disorder” and that they are somehow qualitatively different, as there was not much greater variance in the measures in this subgroup which was of a small sample size?

A further reason for the lack of association between depression symptoms and cognition in this study may be that any association is specific to certain cognitive domains. The meta-analysis [7] did suggest that the association was stronger in executive function than in processing speed, something observed previously in a cross-sectional study of older adults where increasing severity of depression was related to lower semantic fluency scores and poorer ability to shift cognitive set, but not to worsening performances on measures of learning and memory, phonemic fluency, or complex problem-solving [3]. Additionally, contradictory results have been found with regard to the relationship between depression severity and speed of information processing [10]. Therefore, it is plausible that the online cognitive tests may not have been sensitive to the cognitive deficits typical of individuals with depression, or those that might change with symptoms improvement. It is important to recognise that a neuropsychological evaluation in a clinical setting is a more comprehensive assessment of cognition and may be more sensitive to cognitive decrement than the brief, unsupervised online assessment used in this study.

There are some study limitations. Our measure of education was binary, and normative data was age-matched only, which may have masked some education-related variability that could affect cognitive performance. Also, this was an educated, computer-literate sample, which may have impacted on the relationships between depressive symptom severity and CVD risk with cognitive performance [33]. As such, they likely had greater cognitive reserve (usually assessed by educational attainment or occupational complexity) and thus have had some ‘buffer’ against cognitive decline due to depressive symptoms or CVD, although despite this they performed below an aged matched normative sample. Previous studies have shown that higher rates of cognitive reserve protect against deficits in verbal fluency [34] and verbal memory [35] in individuals with elevated symptoms of depression. The use of tools such as *Cogstate* in community-based research or primary care settings will be subject to similar selection bias compared to intensive testing in secondary or tertiary care samples. The same is also true of the attrition, whereby more of those we are most interested in following, i.e. with poorer cognition, were lost to follow-up, although aging in only one of the three domains. For many outcomes loss of the most severe might bias results to the null but with cognition, which tends to show a consistent decline over time, this may not be true. Further, this attrition may limit generalisability but does not explain the lack of association between depressive symptoms and cognitive function in this large sample. Finally, the measures of depressive symptoms [29] and cognition [27, 36, 37] are well-validated although our classification of probable MDD was based upon self-report [29] rather clinical interview, potentially leading to a misclassification bias, although more likely random misclassification. Nevertheless, it may be worthwhile to examine these relationships further in a more clinically robust sample of diagnosed patients with CVD and depression.

### Conclusions

This large study of cognitive function suggests that even when technologically literate and well-educated, adults with mild-to-moderate depression and CVD risks in the community have poorer cognitive function than well peers, particularly in relation to psychomotor speed and learning. However, we were unable to find any of the hypothesised cross sectional or prospective associations of depressive symptoms and cognition in this community-based sample with milder depressive symptoms. This suggests that any such associations may be present only in those with actual MDD as seen in such secondary care samples. As such, alleviating depressive symptoms may lead to improvement in cognitive function only among those with probable MDD (as opposed to more mild symptoms); however, large-scale efforts to prevent progression from more mild symptoms to diagnosed MDD still seem worthwhile as a means to reduce the risk of future cognitive decline [38, 39].

## Supporting information

S1 Data De-identified data set. (SAV)

S2 Data Marginal distribution of outcomes and tests of assumptions. (DOCX)
