# Supplementary material for: The relationship between depression and cognitive function in adults with cardiovascular risk: Evidence from a randomised attention-controlled trial
Source: PLoS One. 2018 Sep 5;13(9):e0203343. doi: 10.1371/journal.pone.0203343 (PMC6124758; doi:10.1371/journal.pone.0203343)
Supplement: S2 Data — (DOCX) [file pone.0203343.s002.docx]

**MARGINAL DISTRIBUTION OF VARIABLES OF INTEREST (and residuals where relevant)**

**COGNITIVE FUNCTION (raw).**

Psychomotor speed BL (n = 441)


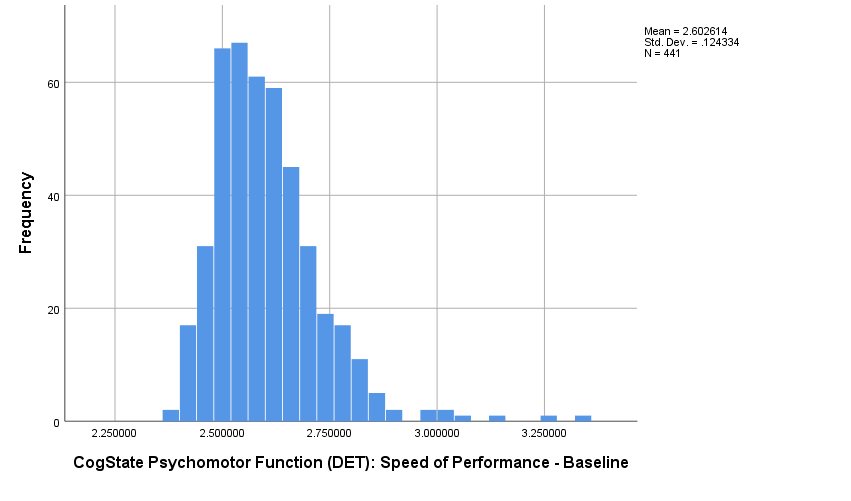


Psychomotor speed (*n = 6 outliers removed*) BL


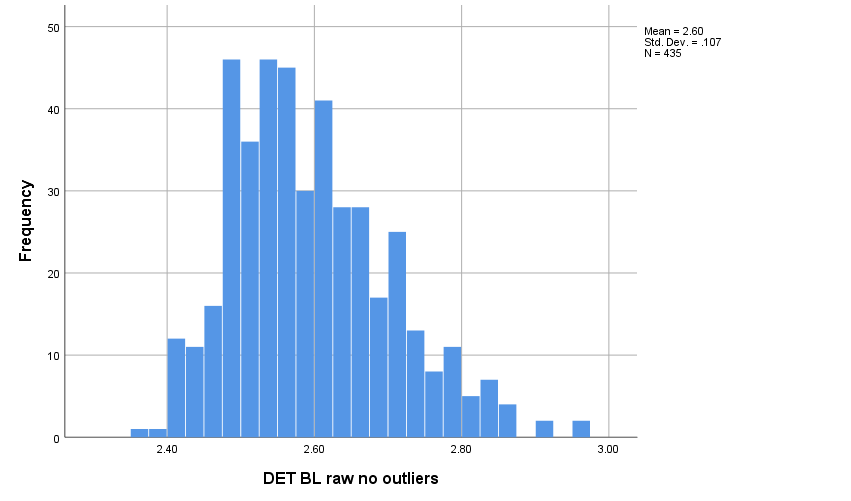


**COGNITIVE FUNCTION (raw).**

Visual learning and memory BL (n = 445)


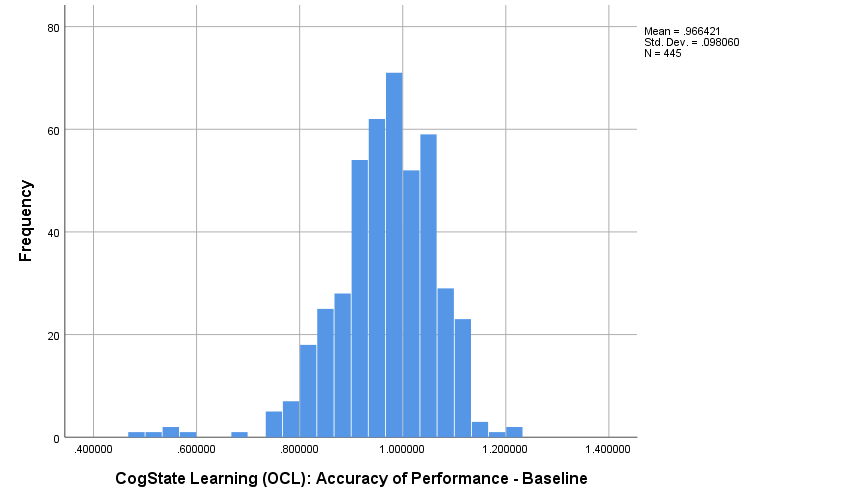


Visual learning and memory (*n = 5 outliers removed*) BL


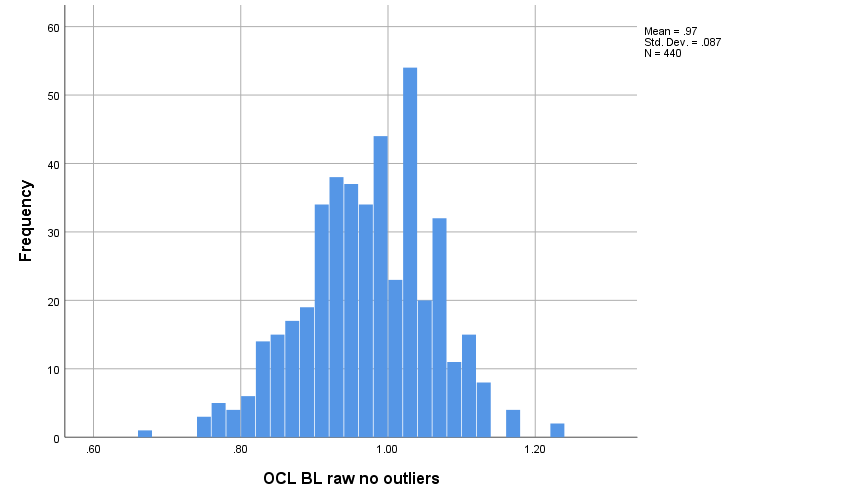


**COGNITIVE FUNCTION (raw).**

Executive function BL_LN (n = 445)


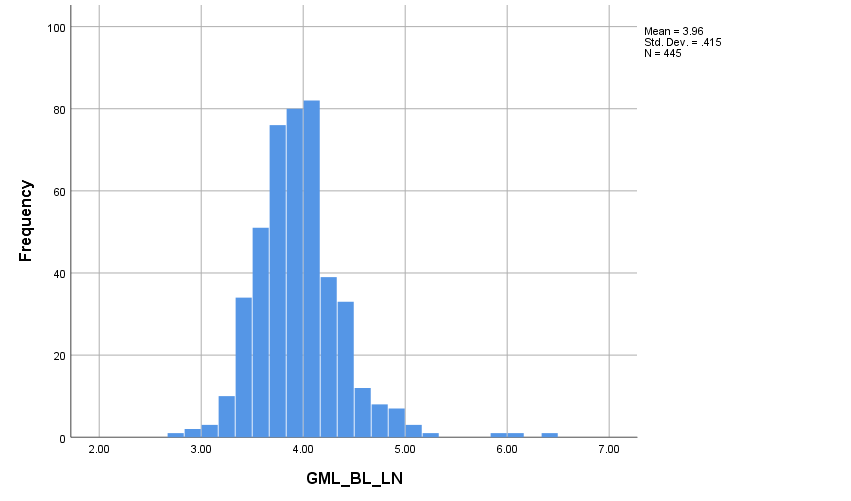


Executive function BL_LN (*n = 3 outliers removed*) BL


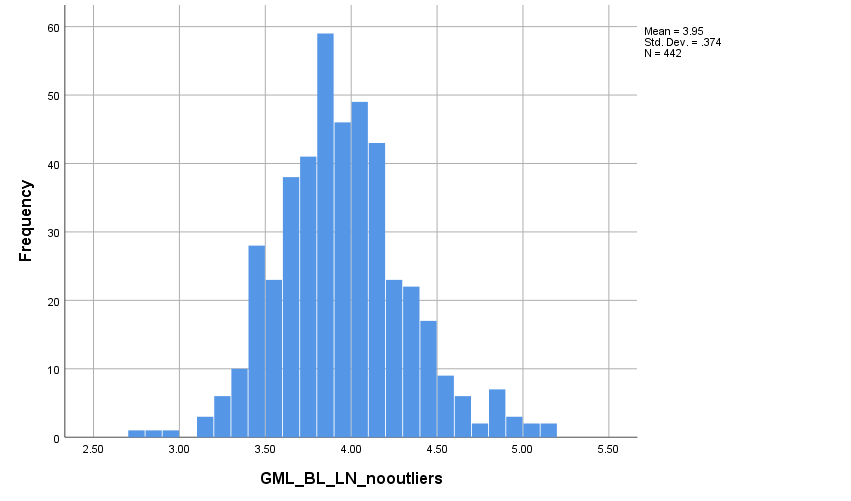


**DEPRESSION (raw).**

Depression BL (PHQ-9 continuous) (n = 445)


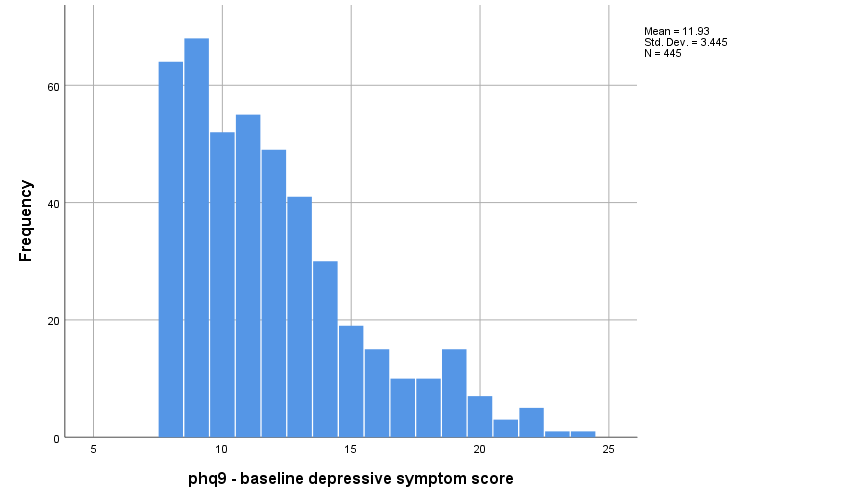


Depression BL (PHQ-9 continuous, n = 2 outliers removed)


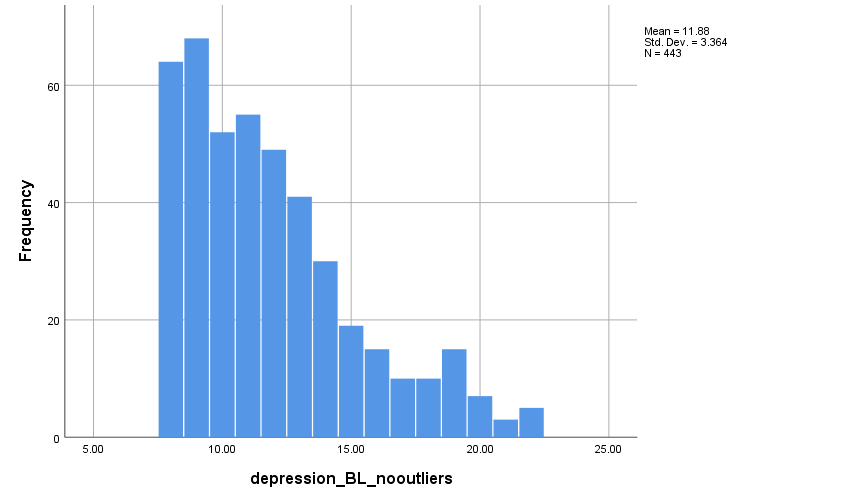


**COGNITIVE FUNCTION (normed). FOR MULTIVARIATE ANALYSES**

Psychomotor speed (*normed*) BL (n = 441)


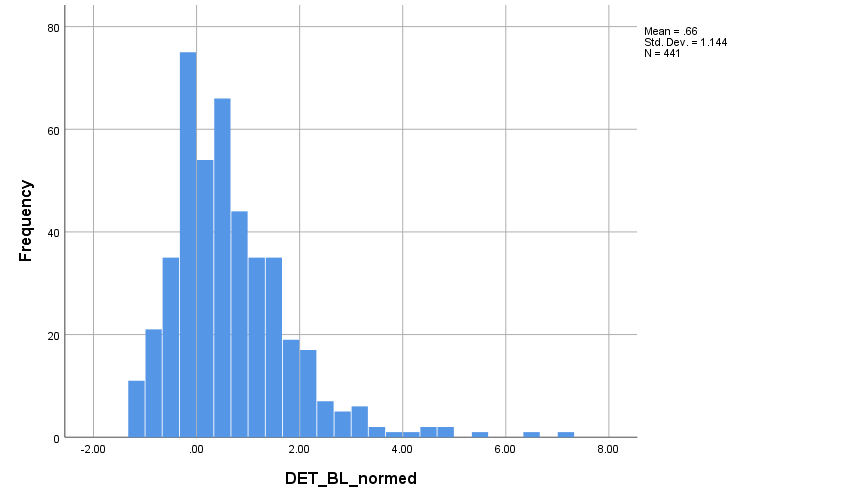


Psychomotor speed (*normed, n = 8 outliers removed*) BL


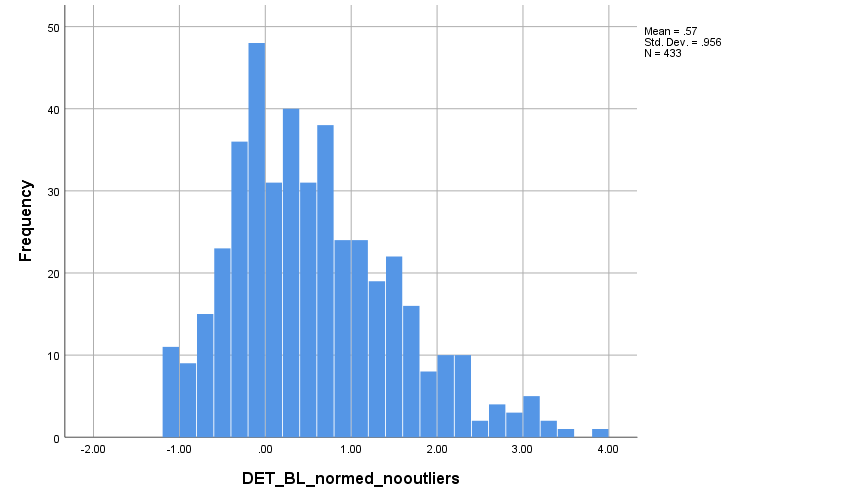


**COGNITIVE FUNCTION (normed). FOR MULTIVARIATE ANALYSES**

Visual learning and memory (*normed*) BL (n = 445)


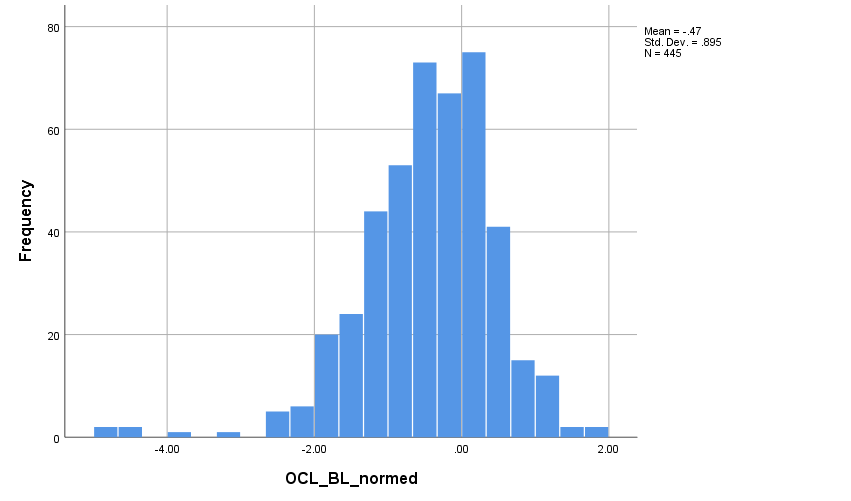


Visual learning and memory (*normed, n = 5 outliers removed*) BL


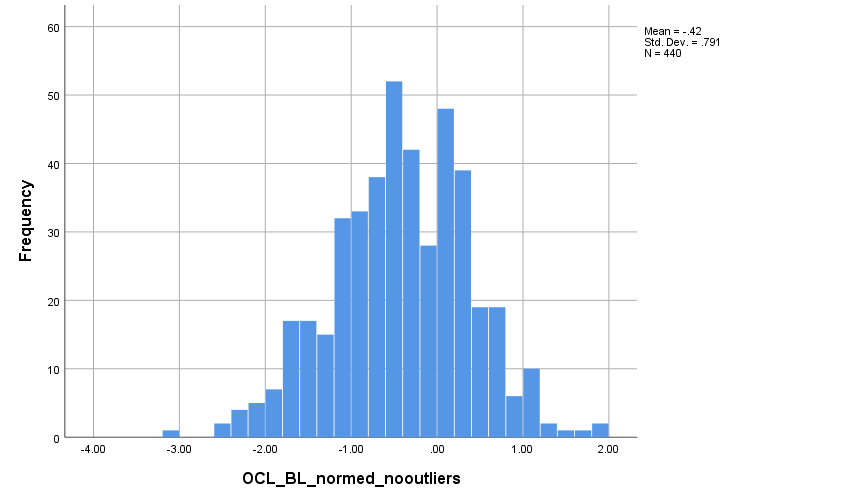


**COGNITIVE FUNCTION (normed). FOR MULTIVARIATE ANALYSES**

Executive function (*normed*) BL_LN (n = 445)


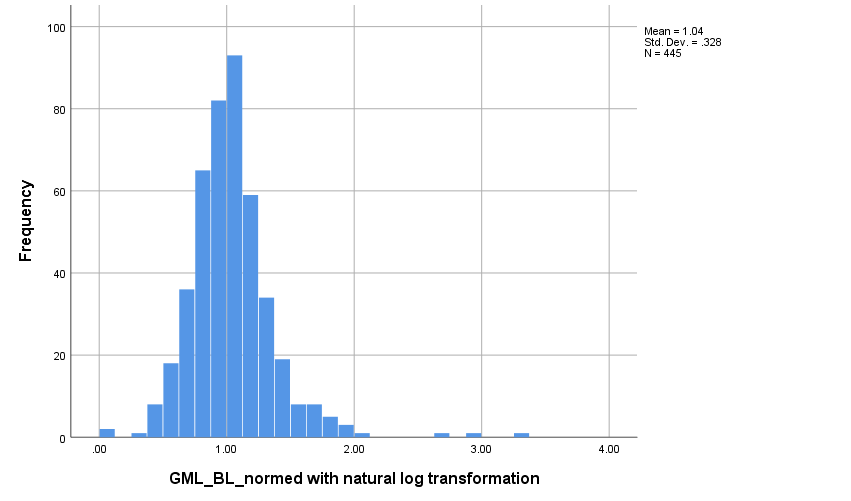


Executive function (*normed, n = 3 outliers removed*) BL_LN


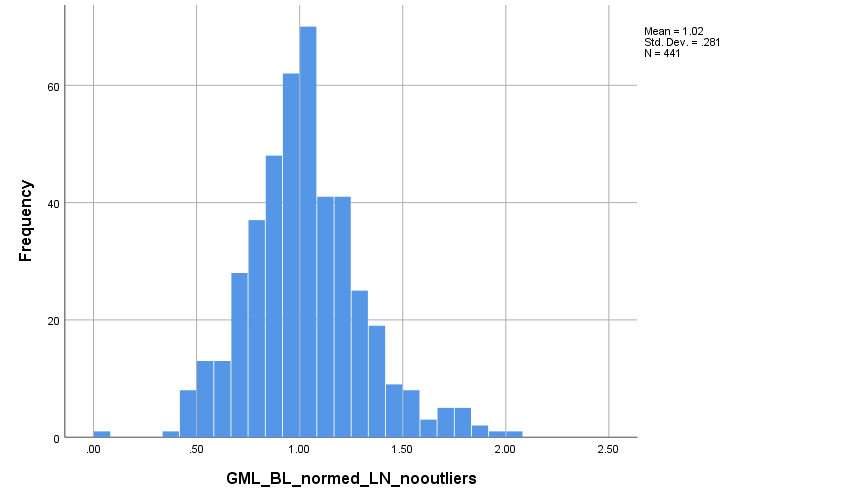


**TABLE 3 PAIRED T-TESTS - DISTRIBUTION OF CHANGE SCORES (change in normed scores for cognition and raw scores for depression).**

Change in psychomotor speed


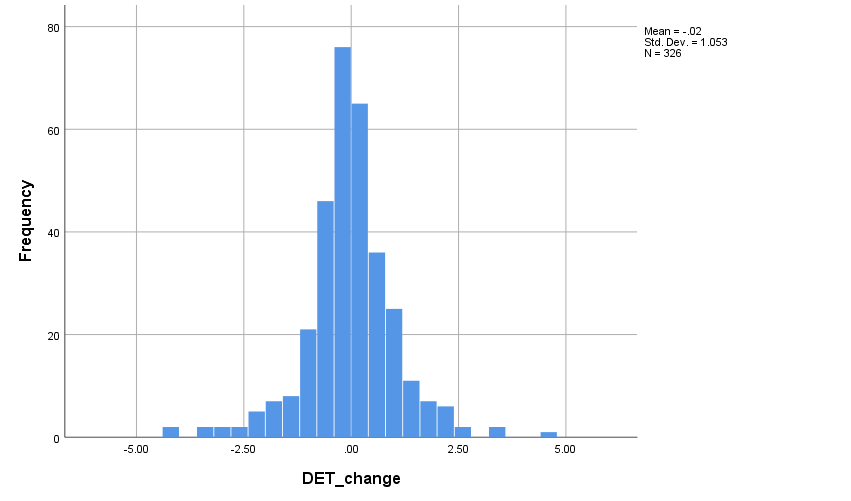


Change in psychomotor speed, n = 7 outliers removed


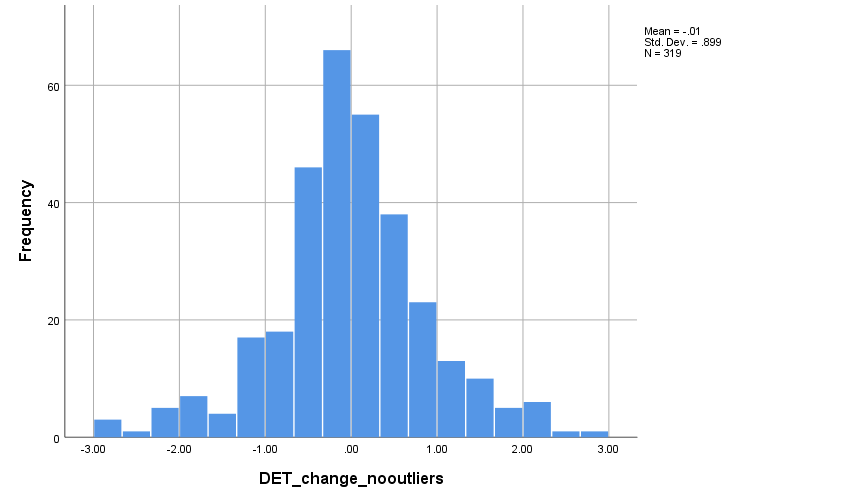


**TABLE 3 PAIRED T-TESTS - DISTRIBUTION OF CHANGE SCORES (change in normed scores for cognition and raw scores for depression).**

Change in visual memory and learning


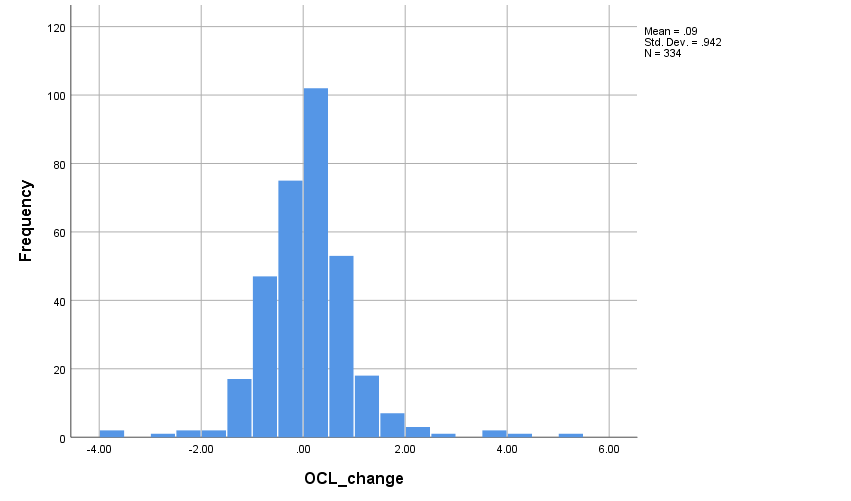


Change in visual memory and learning, n = 7 outliers removed


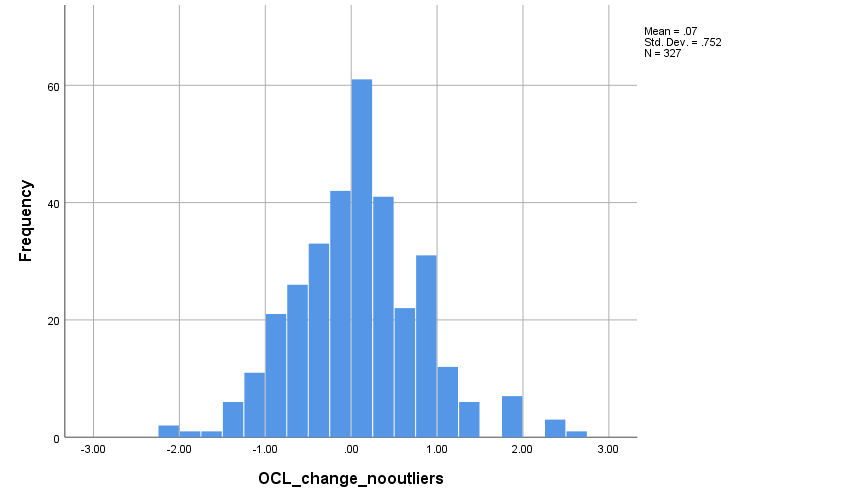


**TABLE 3 PAIRED T-TESTS - DISTRIBUTION OF CHANGE SCORES (change in normed scores for cognition and raw scores for depression).**

Change in executive function


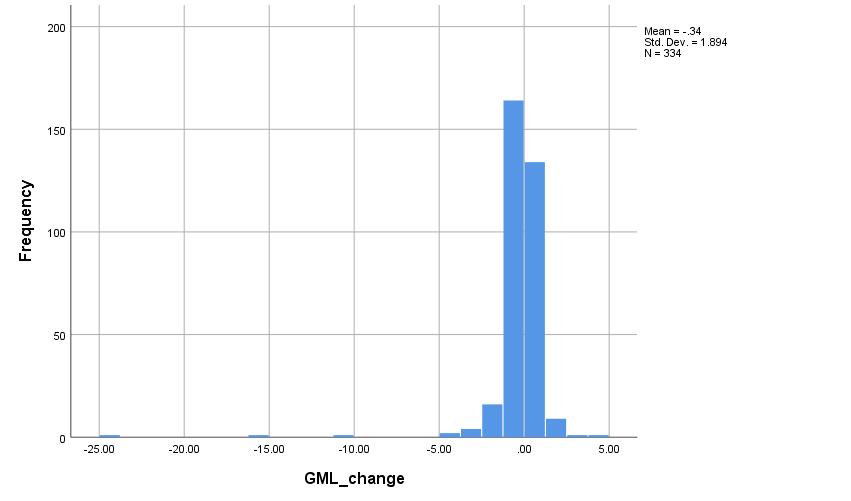


Change in executive function, n = 3 outliers removed


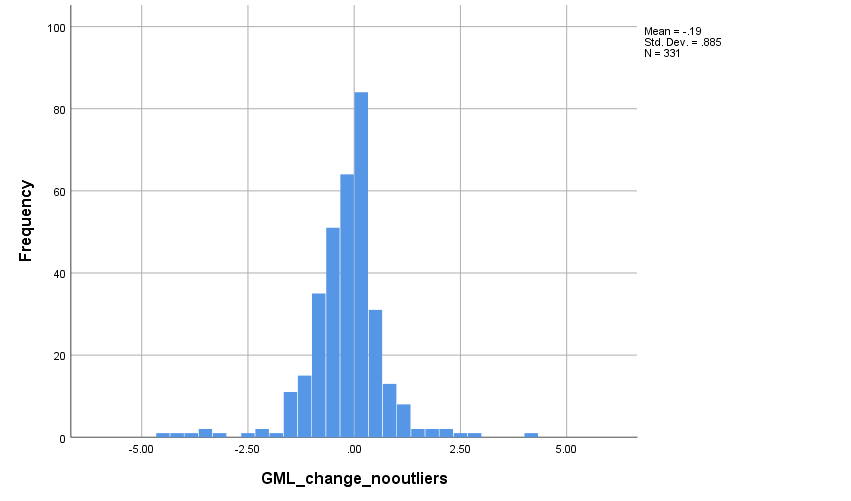


**TABLE 3 PAIRED T-TESTS - DISTRIBUTION OF CHANGE SCORES (change in normed scores for cognition and raw scores for depression).**

Change in depression


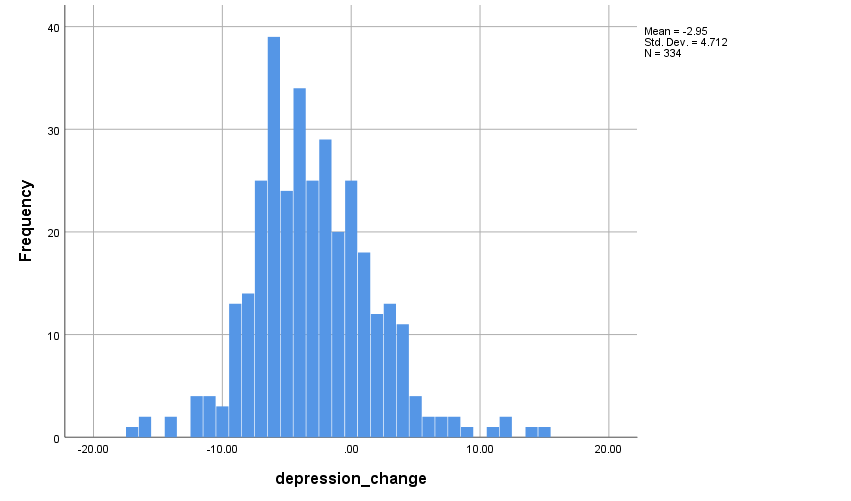


Change in depression, n = 4 outliers removed


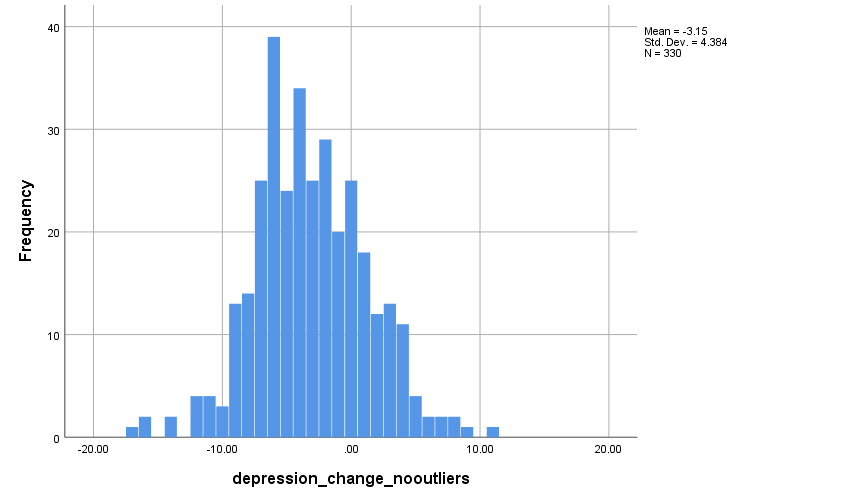


**TABLE 4 CROSS-SECTIONAL LINEAR MODELS. DISTRIBUTION OF STANDARDIZED RESIDUALS.**

Standardized residuals – psychomotor speed regressed on fully-adjusted depression (n=440)


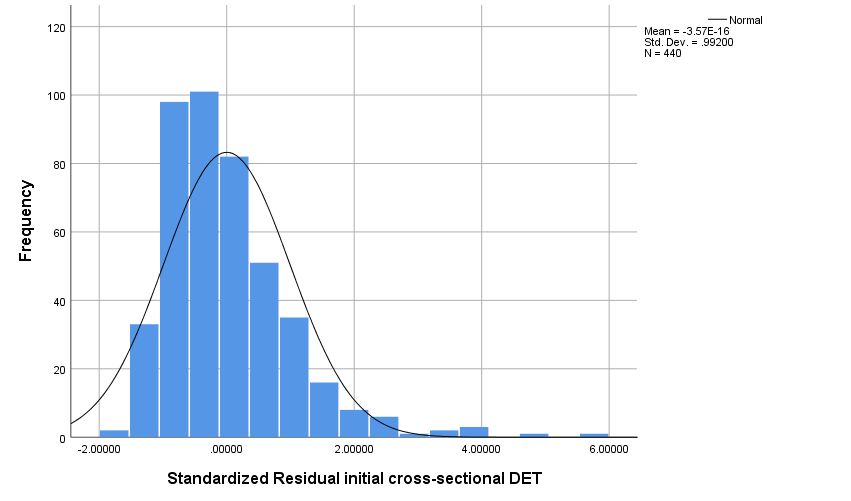


Standardized residuals – psychomotor speed regressed on fully-adjusted depression, (n=8) outliers removed


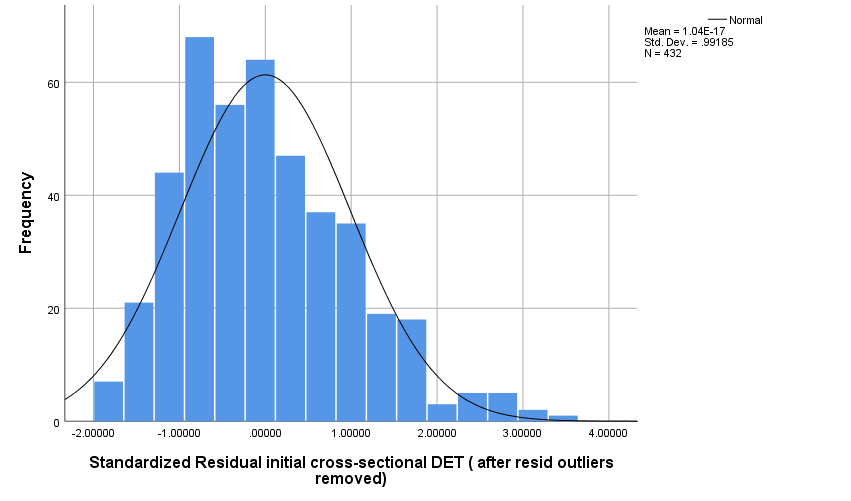


**TABLE 4 CROSS-SECTIONAL LINEAR MODELS. DISTRIBUTION OF STANDARDIZED RESIDUALS.**

Standardized residuals – visual learning and memory regressed on fully-adjusted depression (n=444)


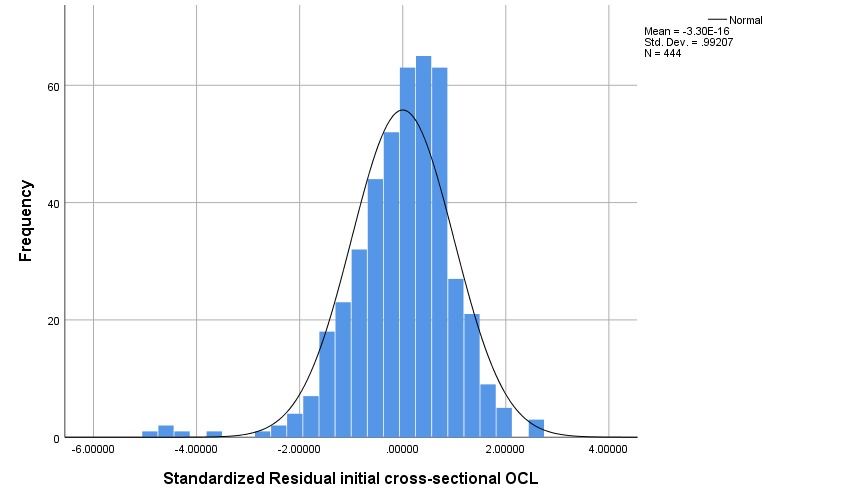


Standardized residuals – visual learning and memory regressed on fully-adjusted depression, (n=5) outliers removed


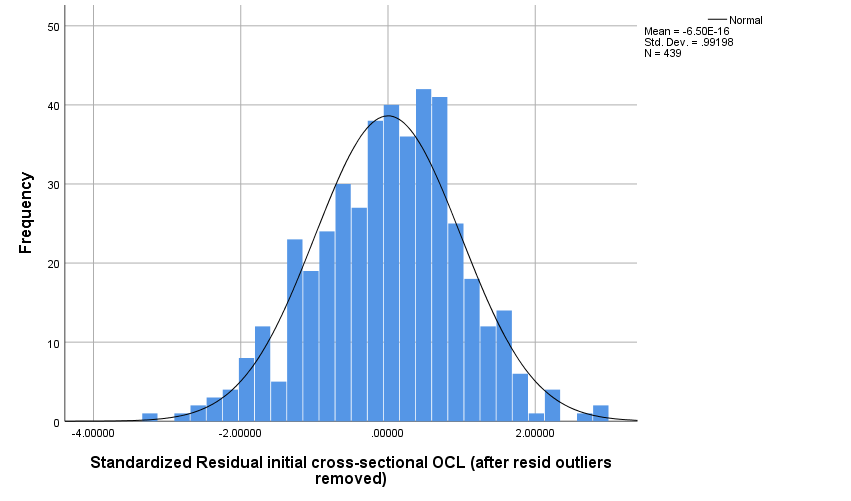


**TABLE 4 CROSS-SECTIONAL LINEAR MODELS. DISTRIBUTION OF STANDARDIZED RESIDUALS.**

Standardized residuals – executive function regressed on fully-adjusted depression (n=444)


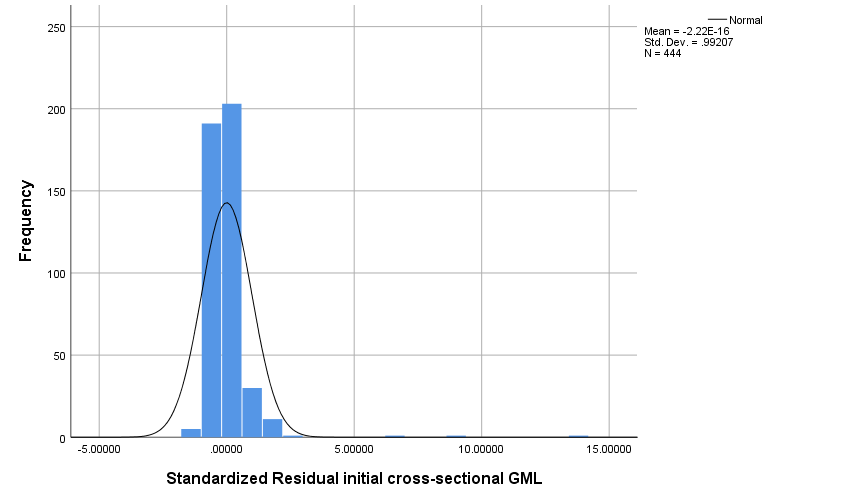


Standardized residuals – executive function regressed on fully-adjusted depression, (n=3) outliers removed


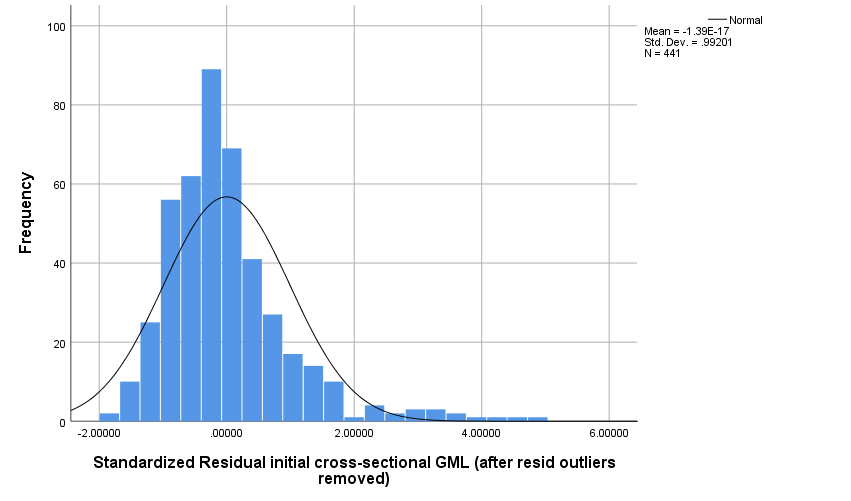


NB distribution of standardized residuals for GML appeared excessively non-normal, even with outliers removed, and so we log-transformed the GML_normed variable. Before this we added the lowest value +1 to every score, to ensure that every value was greater than 0. This resulted in the following distribution of standardized residuals:

Standardized residuals – executive function regressed on fully-adjusted depression (n=444)


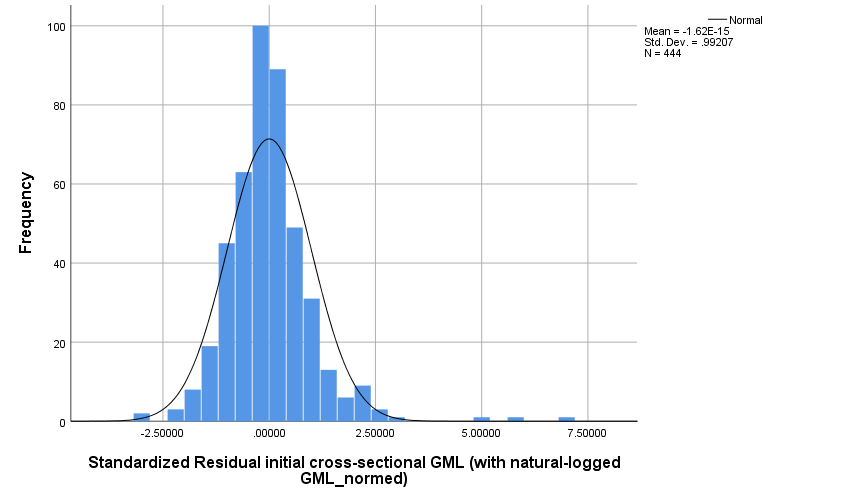


Standardized residuals – executive function regressed on fully-adjusted depression, (n=5) outliers removed
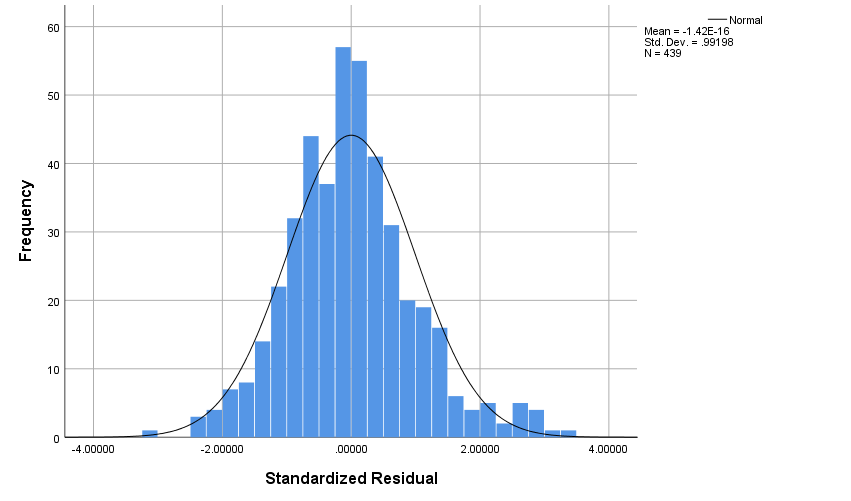


**TABLE 4 CROSS-SECTIONAL MODELS - TESTING NON-LINEARITY and homogeneity of errors. Bivariate plot of the predicted value against residuals with Loess line fitted (to help us infer whether the relationships of the predictors to the outcome is linear). Loess line roughly linear and fitted to 0 suggests assumption met. Homogeneity of variance suggested by equal spread of errors along 0.**

**DET**


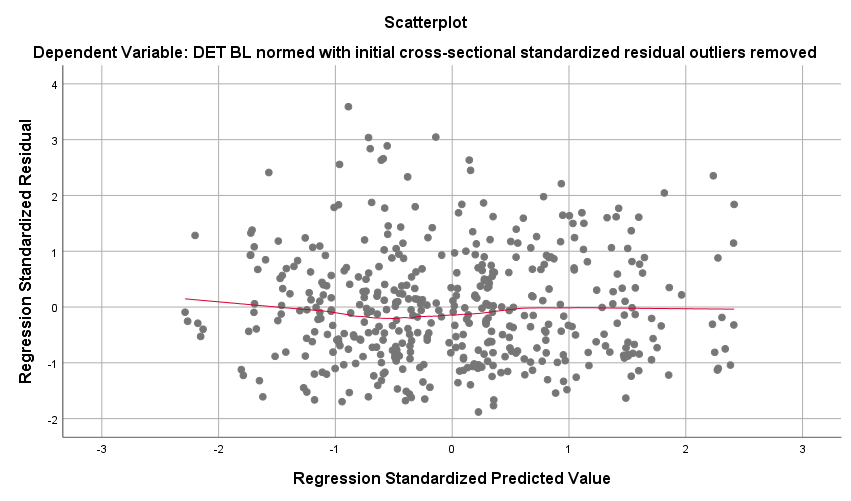


**OCL**


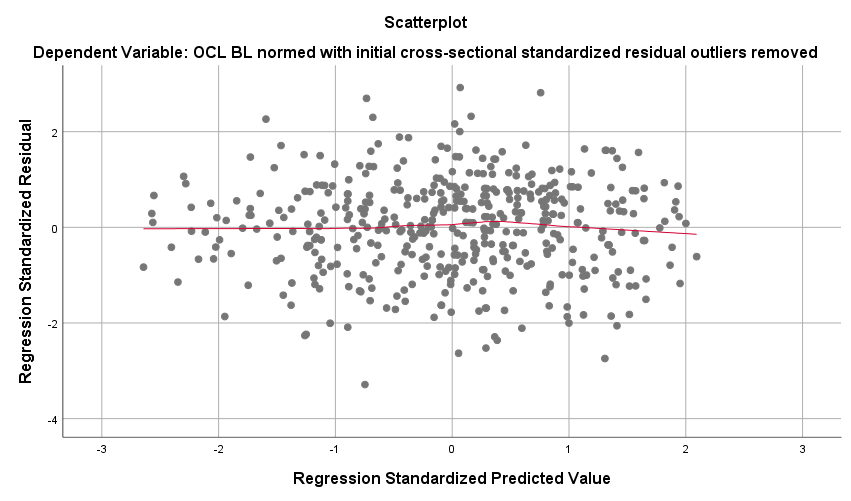


**GML**


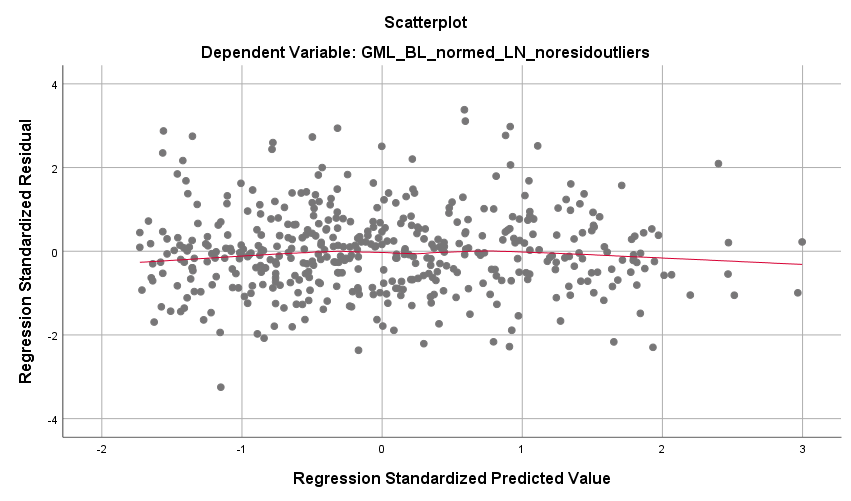


**TABLE 4 CROSS-SECTIONAL MODELS – CONFIRMING LACK OF ASSOCIATION BETWEEN IVs and RESIDUALS**

**
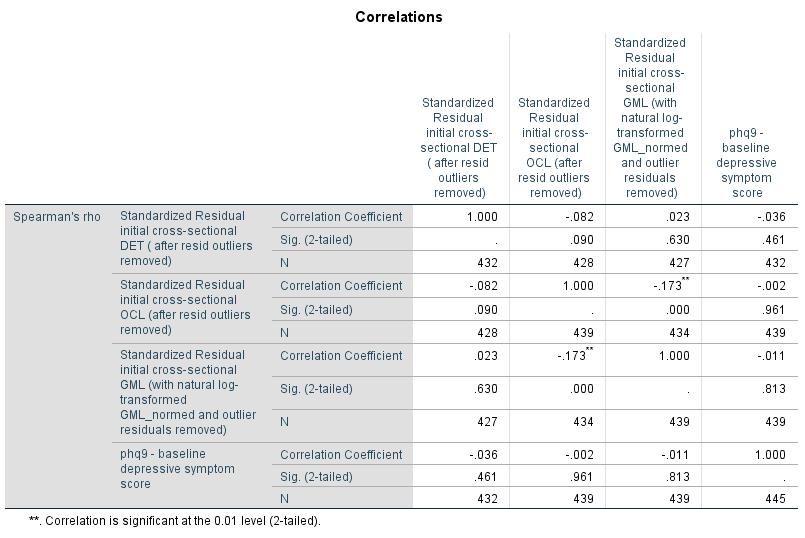
**

No association of residuals with depression, and t-tests confirmed no association of residuals with binary variables.

**TABLE 4 CROSS-SECTIONAL MODELS – CHECKING MULTICOLLINEARITY (VIF values > 10 potentially problematic)**

DET

No VIF values greater than 1.071 at any step.

OCL

No VIF values greater than 1.063 at any step.

GML

No VIF values greater than 1.062 at any step.

**TABLE 4 CHANGE MODELS. DISTRIBUTION OF STANDARDIZED RESIDUALS.**

Standardized residuals – change in psychomotor speed regressed on change in depression, fully-adjusted model (n=325)


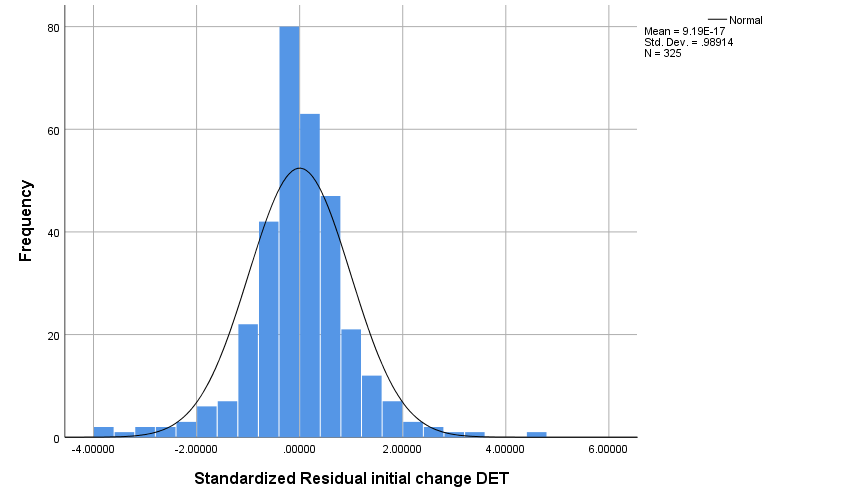


Standardized residuals – change in psychomotor speed regressed on change in depression, fully-adjusted model (n=7 outliers removed)


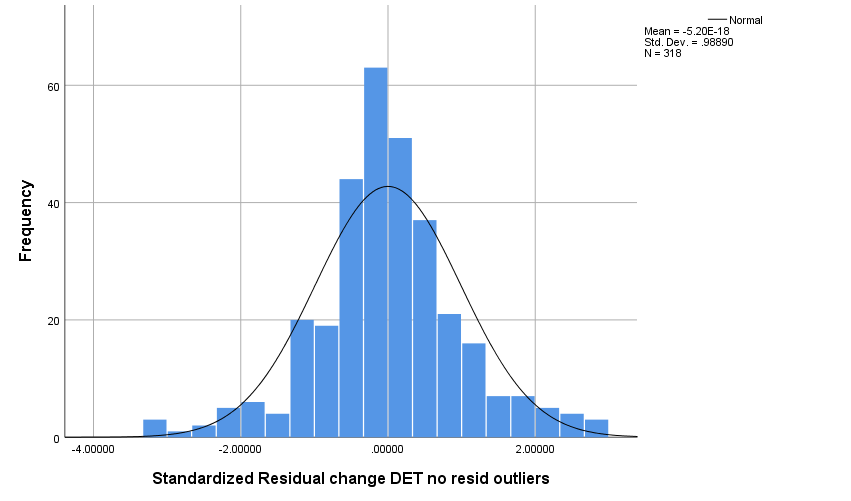


**TABLE 4 CHANGE MODELS. DISTRIBUTION OF STANDARDIZED RESIDUALS.**

Standardized residuals – change in learning and memory regressed on change in depression, fully-adjusted model (n=333)


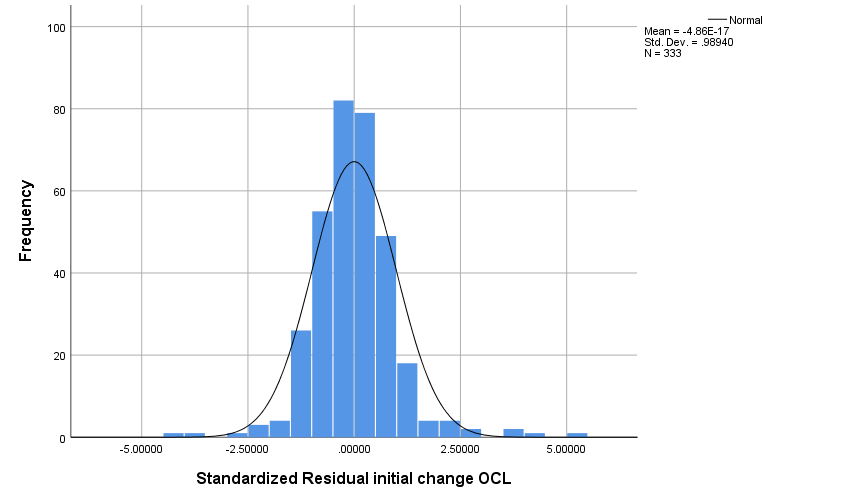


Standardized residuals – change in psychomotor speed regressed on change in depression, fully-adjusted model (n=6 outliers removed)


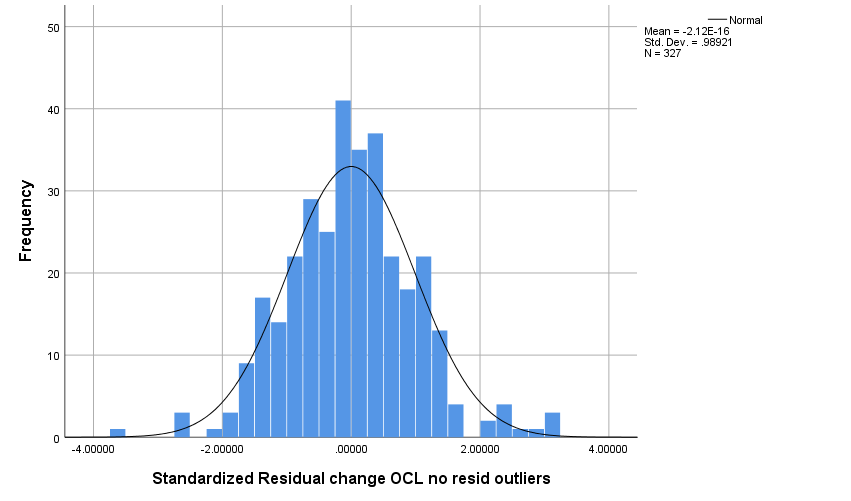


**TABLE 4 CHANGE MODELS. DISTRIBUTION OF STANDARDIZED RESIDUALS.**

Standardized residuals – change in executive function regressed on change in depression, fully-adjusted model (n=333)


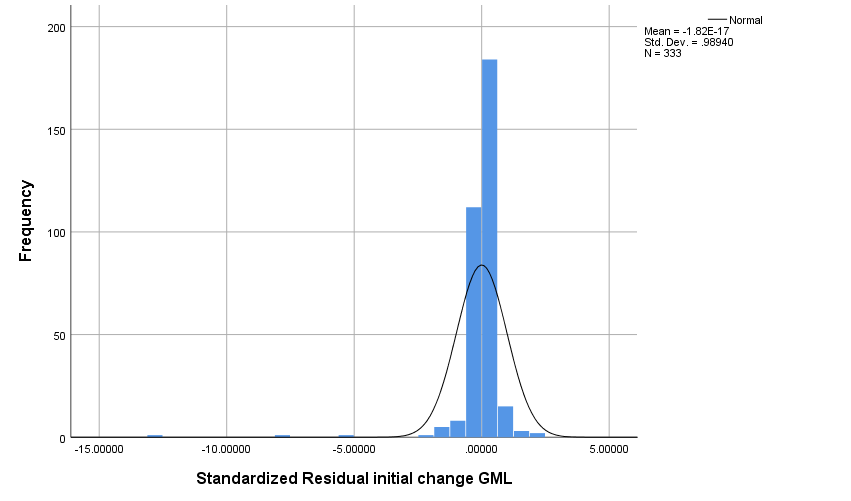


Standardized residuals – change in executive function regressed on change in depression, fully-adjusted model (n=3 outliers removed)


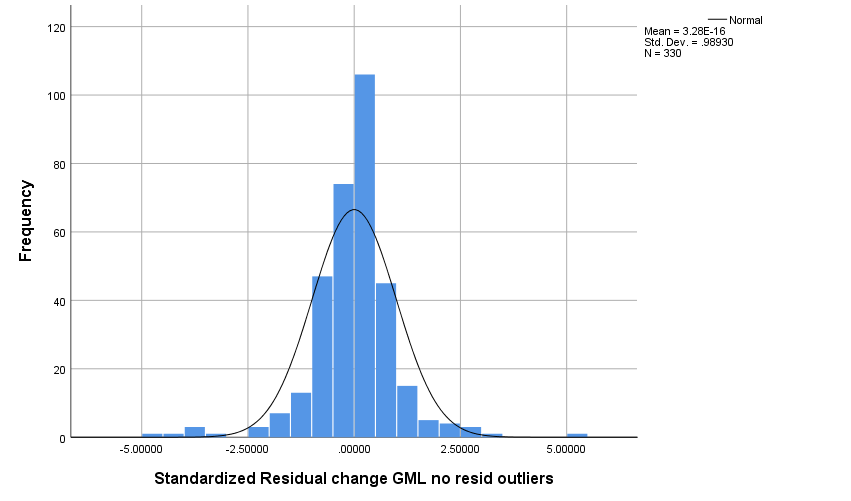


**TABLE 4 CHANGE MODELS - TESTING NON-LINEARITY AND HOMOGENEITY OF ERRORS. Bivariate plot of the predicted value against residuals with Loess line fitted (to help us infer whether the relationships of the predictors to the outcome is linear). Loess line roughly linear and fitted to 0 suggests assumption met. Homogeneity of variance suggested by equal spread of errors along 0.**

**DET**


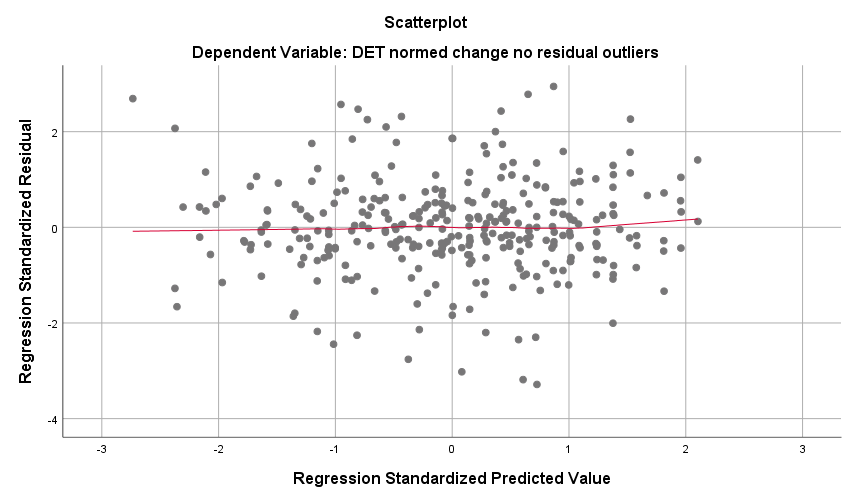


**OCL**


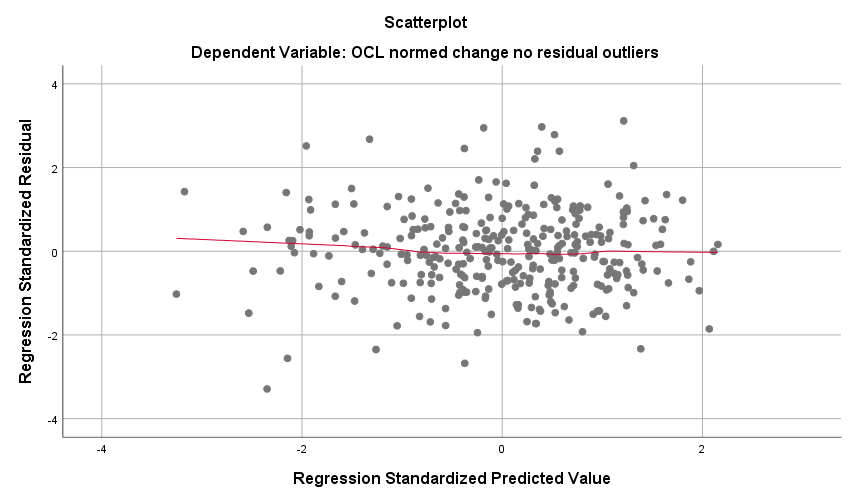


**TABLE 4 CHANGE MODELS - TESTING NON-LINEARITY AND HOMOGENEITY OF ERRORS (CONTINUED). Bivariate plot of the predicted value against residuals with Loess line fitted (to help us infer whether the relationships of the predictors to the outcome is linear). Loess line roughly linear and fitted to 0 suggests assumption met. Homogeneity of variance suggested by roughly equal spread of errors along 0.**

**GML**


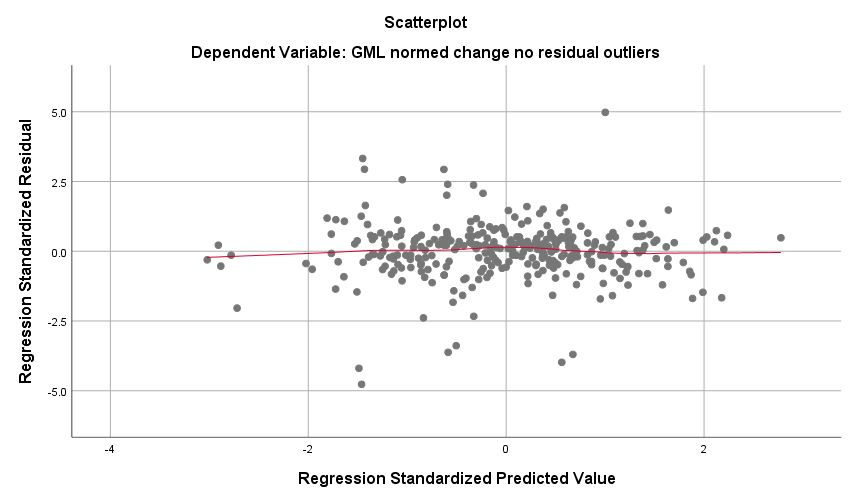


**TABLE 4 CHANGE MODELS – CONFIRMING LACK OF ASSOCIATION BETWEEN IVs and RESIDUALS**

**
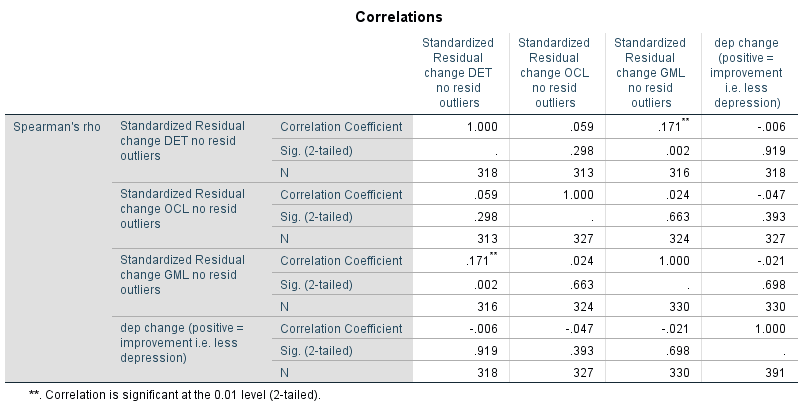
**

No association of residuals with depression change, and t-tests confirmed no association of residuals with binary variables.

**TABLE 4 CHANGE MODELS – CHECKING MULTICOLLINEARITY (VIF values > 10 potentially problematic)**

DET change

No VIF values greater than 1.049 at any step.

OCL change

No VIF values greater than 1.056 at any step.

GML change

No VIF values greater than 1.051 at any step.
